# Natural Killer Cells in the Orchestration of Chronic Inflammatory Diseases

**DOI:** 10.1155/2017/4218254

**Published:** 2017-03-27

**Authors:** Luca Parisi, Barbara Bassani, Marco Tremolati, Elisabetta Gini, Giampietro Farronato, Antonino Bruno

**Affiliations:** ^1^Department of Biomedical, Surgical and Dental Sciences, University of Milan, Milan, Italy; ^2^Scientific and Technological Pole, IRCCS MultiMedica, Milan, Italy; ^3^Department of Biotechnology and Life Sciences (DBSV), University of Insubria, Varese, Italy

## Abstract

Inflammation, altered immune cell phenotype, and functions are key features shared by diverse chronic diseases, including cardiovascular, neurodegenerative diseases, diabetes, metabolic syndrome, and cancer. Natural killer cells are innate lymphoid cells primarily involved in the immune system response to* non-self*-components but their plasticity is largely influenced by the pathological microenvironment. Altered NK phenotype and function have been reported in several pathological conditions, basically related to impaired or enhanced toxicity. Here we reviewed and discussed the role of NKs in selected, different, and “distant” chronic diseases, cancer, diabetes, periodontitis, and atherosclerosis, placing NK cells as crucial orchestrator of these pathologic conditions.

## 1. Introduction

Inflammation is now considered a crucial hallmark of chronic disorders, including cardiovascular [1], neurodegenerative diseases [2–4], diabetes [5, 6], metabolic syndrome [7, 8], and cancer [9–11]. Inflammation acts as a relevant orchestrator in their insurgence, development, and progression [1, 2, 6, 10]. Inflammatory cells, which comprise cells from innate ad adaptive immunity, are characterized by different phenotype and functions, which involve direct (by contact) or distant (by soluble factors) interaction with their target cells [12–14]. Immune cells, form either innate or adaptive immunity, given their cellular plasticity, have been reported to acquire an altered phenotype upon different stimuli. This has been described for diverse immune cell type, including macrophages (M), neutrophils (N), myeloid-derive-suppressor cells (MDSC), dendritic cells (DC), natural killer (NK) cells, and T cells [12–14]. Immune cell altered functions include attenuation of targeting/killing activities, tolerogenic/immunosuppressive behaviour, and the acquisition of proangiogenic functions. These alterations, occurring at both tissue levels and systemically, are finely tuned by the (chronic) pathological environment. Here, we focused on NKs as key orchestrator in inflammatory chronic diseases, such cancer, type 1 diabetes, periodontitis, and atherosclerosis. The aim was to discuss how such very different pathologies, with diverse aetiology and photogenic mechanisms, share a common and relevant hallmark, such inflammation, dissecting whether NK cells act as crucial orchestrators in the induction and progression of the conditions selected.

**Figure 1:**
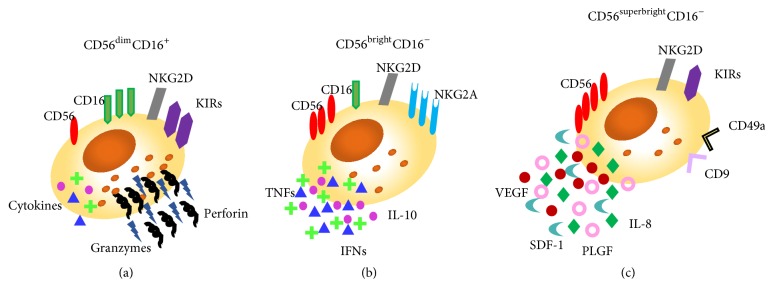
Different human NK subsets. Three different NK subsets have been characterized on the basis of distinctive surface antigen expression and described as (a) CD56^dim^CD16^+^ that represents the conventional cytotoxic NKs, characterized by the high release of perforin and granzymes; (b) CD56^bright^CD16^−^ that are able to release a large amount of cytokines; (c) CD56^superbright^CD16^−^ that are able to produce proangiogenic factors, including VEGF, IL-8, and SDF-1 and are characterized by the expression of specific surface markers as CD9 and CD49a.

NKs are innate lymphoid cells, primarily involved in the host defence against infection and in the process of tumour immunosurveillance. A part from their crucial role in those processes, NK cells are involved in the graft-versus-host disease, the regulation of haematopoiesis, and exert regulatory effects on the adaptive immune cell counterpart [15]. Virus infected and tumour transforming cells share the feature of low/null expression of the MHC-I molecule, representing one of the mechanisms through which NK cells are able to recognize target/*non-self*-cells. Nevertheless, this mechanism alone does not trigger cytotoxicity, unless it is combined with the altered expression of other molecules on the target cell surface, acting as activatory ligands. NK cells are equipped with surface receptors that trigger cell activation (immunoreceptor tyrosine-based activation motifs (ITAM)) or inhibition (immune tyrosine-based inhibitory motifs (ITIM)) [16]. NK cells also directly contribute to adaptive immune responses, interacting with DCs and by triggering T cell responses. Induction of DC maturation to produce TNF-*α* and IL-12 and upregulation of costimulatory ligands are triggered by NK cells [17]. Moreover, NK cells proliferate and acquire cytotoxic activity and the capacity to produce IFN-*γ* through the interaction with DCs [18]. Apart from activation of other cells of innate immunity, NK cells also enhance induction of CD8^+^ T cell responses that is influenced by NK-released IFN-*γ*, which promotes antigen processing and presentation to T cells and T helper type 1 (Th1) cell polarization [19]. Two major circulating human NK cell subsets have been characterized based on the expression of surface antigens: CD56, an isoform of the human neural cell adhesion molecule, and CD16, the low-affinity Fc receptor necessary for the antibody-dependent cellular cytotoxicity (ADCC). Peripheral NK cells are predominantly CD56^dim^CD16^+^ cytotoxic NK cells acting mainly through the release of perforin, a membrane pore-forming toxin, and granzyme which activates the apoptotic cascade on target cells. However, approximately 5% of circulating NK cells show a CD56^bright^CD16^−^ phenotype. These cells can produce high levels of some cytokines. Upon activation, CD56^bright^CD16^−^ NK cells release IFN-*γ* and TNF-*α*, and they kill target cells more efficiently [20]. Within the developing decidua, a third NK subset is found, the decidual NK cell (dNK). dNK cells display a CD56^superbright^CD16^−^ phenotype [21] and are closely linked with vascularization of the decidua in both humans and mice. dNK cells physiologically produce VEGF, PlGF, and IL-8, are poorly cytotoxic, and are associated with the induction of CD4^+^ T regulatory (Treg) cells [22, 23] (Figure 1).

## 2. Natural Killer Cells and Cancer

Strong evidences suggest that the presence of inflammatory cells within the tumour microenvironment (TME) plays a crucial role in the development and/or progression of human cancers [10, 12, 24–27]. Among the host-dependent biological features of the tumour hallmarks, defined by Hanahan and Weinberg [28], there are “evading immune destruction” and “tumour-promoting inflammation,” which together with the immune orchestration of angiogenesis point out the key role of the immune system in neoplastic diseases [9, 10, 12, 24].

Alterations in NK cell activity have been described in different type of cancer and are associated with the induction of a tolerogenic and less/poor cytotoxic functions with decreased expression of the activatory receptor NKG2D, altered degranulation, and release of perforin and granzyme. Recently, Bruno et al. identified a new NK cell subset in tissue and peripheral blood of non-small-cell lung cancer (NSCLC) patients, termed, respectively, tumour infiltrating (TINKs) and tumour associated (TANKs) NKs, which are able to promote angiogenesis [29, 30]. NSCLC TINK/TANKs are characterized by a decidual like phenotype CD56^bright^CD16^−^VEGF^high^PlGF^high^IL-8^+^IFN-*γ*^low^, able to promote endothelial cell migration and induction of capillary-like structures [29, 30].

**Figure 2:**
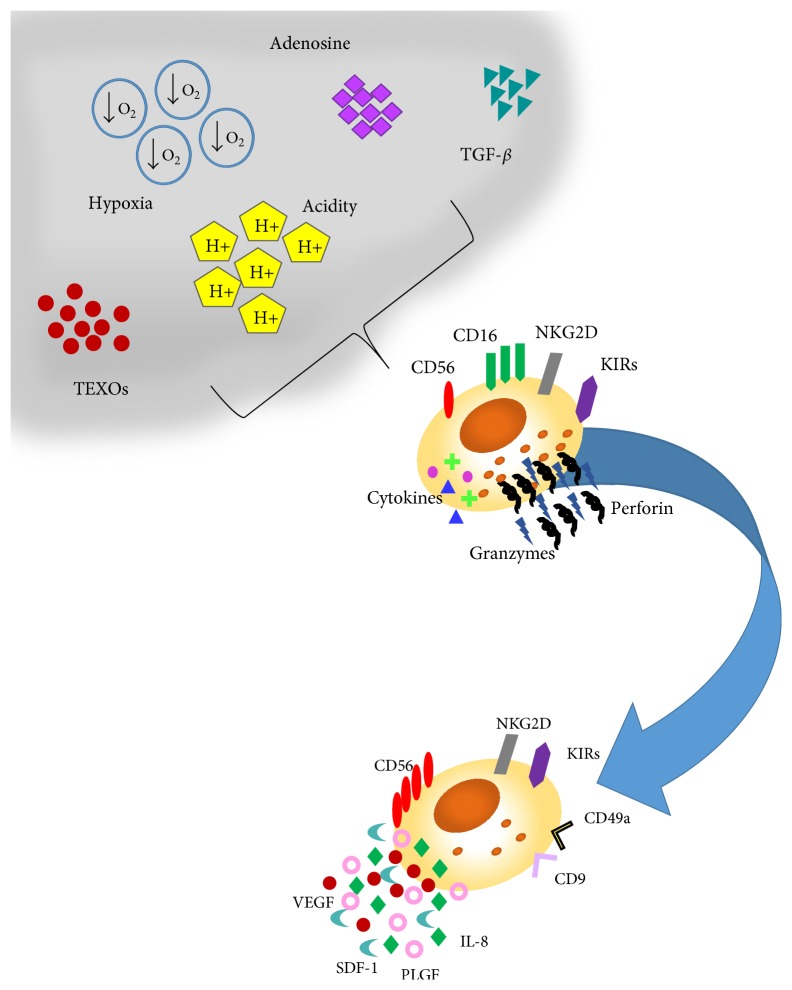
Soluble factors within the tumour microenvironment shaping NK cell functions. Several molecules and soluble factors within the tumour microenvironment, including TGF-*β*, hypoxia, adenosine, acidic of the environment, and tumour exosomes (TEXOs), can inhibit NK response against tumours either by interfering with NK cell direct/cytokine mediated tumour cell lysis or by supporting tumour angiogenesis.

Several TME released components, including TGF-*β*, hypoxia, and adenosine, mostly shared with the decidual tissues, are implicated in NK cell response against tumours [31] (Figure 2).

TGF-*β* is one of the numerous TME factors involved in the induction of immune cell polarization [32] and is expressed at high levels both in the tumour microenvironment and in the decidua [29]. During carcinogenesis, TGF-*β* acts as a tumour suppressor, by inhibiting tumour cell replication and favouring apoptosis [33, 34], while at later stages of tumour progression it exerts protumourigenic effects that include tumour survival, induction of epithelial-mesenchymal transition (EMT), enhanced tumour invasion, and immunosuppressive and proangiogenic activities [32–34]. TGF-*β* has been found to polarize the CD56^dim^CD16^+^ peripheral NK cells towards a decidual like phenotype, defined as CD56^bright^CD16^−^ and KIR^+^ CD9^+^ CD49a^+^ [29, 35–37]. TGF-*β* has been shown to inhibit CD16 mediated human NK cell IFN-*γ* production and ADCC though SMAD3 [36]. Bruno et al. demonstrated that TGF-*β* significantly contributes in the induction of the angiogenic-switch of NK cells from healthy individuals [30], promoting the induction of the TINK/TANK CD56^bright^CD16^−^VEGF^high^PlGF^high^IL-8^+^INF*γ*^low^ phenotype in vitro.

A hypoxic microenvironment is another common feature shared between the decidua and the TME [38, 39]. A combination of TGF-*β* hypoxia and 5-aza-2′-deoxycytidine, a demethylating agent, has been found to convert FACS sorted peripheral blood CD56^dim^CD16^+^NK cells into dNKs, characterized by low cytotoxicity and high expression levels of VEGF, the CD9 dNK marker, and KIRs [36].

Adenosine is a soluble immunomodulatory molecule acting through adenosine receptors expressed on diverse immune cell type, including NK cells [40, 41]. Up to 20-fold increases in the adenosine content in extracellular fluid of solid carcinomas have been reported [42]. Adenosine accumulation is partially associated with hypoxia and its release in the extracellular environment and can impair NK cell cytolytic activities by decreasing TNF-*α* secretion (following IL-2 stimulation), decreasing cytotoxic granule exocytosis, and attenuating perforin and Fas ligand-mediated cytotoxic activity as far as cytokine release. Most of these effects are attributed to stimulation of the cyclic adenosine monophosphate/protein kinase A (PKA) pathway, following the binding of adenosine to A2A receptors on NK cells [43].

Recently, great interests arise on tumour released vesicles, including exosomes, in shaping immune cell response [44, 45]. Exosomes are small (40 to 110 nm) membrane vesicles of endocytic origin which are actively secreted from several cell types. Exosome content includes a variety of biologically active molecules such as proteins, mRNAs, and miRNAs reflecting the cell of origin. They probably mediate a range of local and systematic functions, including immune stimulation or suppression, cell-to-cell communication, delivery of proteins, and genetic material, including miRNA, tumour immune escape, and tumour cell communication [46, 47]. Tumour derived exosomes appear to regulate NK cells impairing their killing activity by downregulating perforin/granzyme production and/or NKG2D ligand expression [48, 49]. Exosome release could explain the effects of tumours on the polarization of peripheral NK cells towards TANK phenotype. The NKG2D/NKG2DL system plays an important role in tumour immune surveillance [42, 48, 49]. There are convincing evidences that exosomes derived from diverse cancer cell lines, including mesothelioma, breast, and prostate cancer cells, express NKG2D ligands, and thereby downregulate NKG2D expression on NK cells and CD8^+^ T cells, resulting in impaired cytotoxic effector functions [48–50]. It has also been shown that leukaemia/lymphoma T and B cells secrete NKG2D ligand-expressing exosomes with the ability to impair the cytotoxic potency of NK and T cells from healthy donors [44, 45].

Recently, STAT5 has been proposed as a key regulator in NK cells and demonstrated that STAT5 acts as a molecular switch from tumour surveillance to tumour promotion [39]. Consistent with its function as the major STAT protein downstream of IL-7, IL-2, and IL-15, Gotthardt et al. reported STAT5 role in tumour angiogenesis showing that* Stat5*^Δ/Δ^*Ncr1*-iCre^Tg^-*Vav-Bcl2 *mice displayed an increased tumour growth compared with wild-type mice [51]. In addition, production of VEGF by NK cells is higher in STAT5-deficient mice compared with wt-mice. To elucidate the role of VEGF production in NKp46^+^ cells, Gotthardt and colleagues established Vegfa^Δ/Δ^Ncr1-iCre^Tg^ mice, characterized by NKp46^+^VEGF^−^ cells. In v-abl^+^ tumour, RMA-S, and A-MuLV-induced leukaemia tumour models, they showed a significant reduction of tumour burden and fewer CD31^+^ blood vessels in tumours.

## 3. Natural Killer Cells in Type 1 Diabetes

Type 1 diabetes (T1D), an autoimmune disease characterized by almost complete beta cell destruction and hyperglycaemia, accounts for only about 5–10% of all cases of diabetes, whereas its incidence is dramatically increasing worldwide over the last 50 years [52]. Different immune cells, such macrophages and dendritic and T cells, have been suggested to play crucial roles in type 1 diabetes pathogenesis [53–55]. The contribution of autoreactive T cells to the destruction of pancreatic *β* cells as a consequence of an immunologically mediated destruction of the pancreatic tissues has been proposed as the key pathogenic mechanisms in type 1 diabetes [56, 57]. Nevertheless, diverse inflammatory cells, from both innate and adaptive immunity, interact with the pancreatic parenchyma, supporting the overall inflammatory state in T1D. NKs cells represent the major source of IFN-*γ*, a Th1 proinflammatory cytokine acting as a master regulator of different immune cell response. High release of IFN-*γ* within the pancreatic tissues in T1D patients may significantly contribute to the excessive, uncontrolled, and unresolved autoimmune response mediated by autoreactive T cells. While NK cell response against autologous pancreatic islet has been reported in vitro [58], contrasting results have been reported in in vivo models.

**Figure 3:**
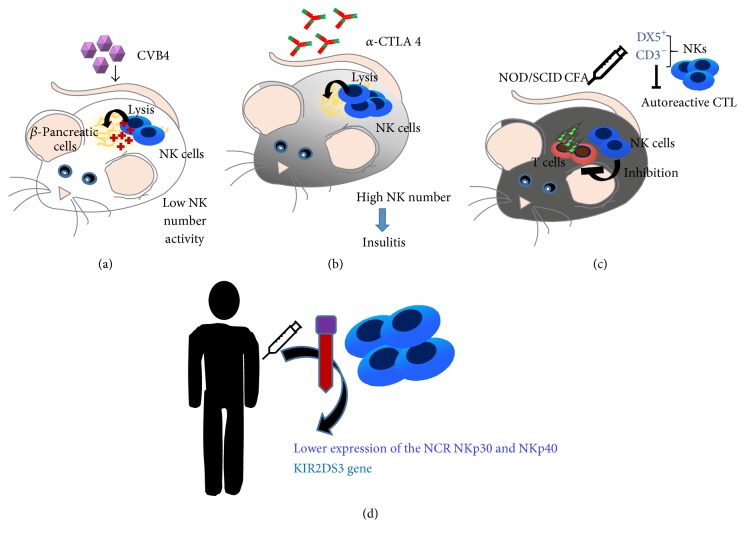
Natural killer cells in type1 diabetes. Several in vivo studies correlate NK cells to diabetes progression: an in vivo model of coxsackievirus B4- (CVB4-) induced diabetes showed that NK antiviral defence resulted in a reduced permissiveness to infection and subsequent NK cell-dependent death (a). Anti-CTLA-4 mAb treatment of T cell receptor transgenic mice demonstrated that higher frequency of NK cells induces aggressive insulitis, resulting in b-islet cell destruction (b). A protective role of NK cells was reported in NOD mice undergoing complete Freund's adjuvant (CFA). NOD/SCID mice immunized with CFA recover its protective effects when CD3^−^DX5^+^ NKs are adoptively transferred into animal recipients, by downregulating autoreactive T cell response (c). In human samples, lower expression of the NKp30 and NKp40 was detected in type 1 diabetic patients as compared with control. Type 1 diabetic patients display an increased frequency of KIR gene haplotypes, including the activating KIR2DS3 gene, with a genetic interaction between the KIR and HLA complexes (d).

Two in vivo studies correlate NK cells to diabetes progression. In the first study (Figure 3(a)), an in vivo model of coxsackievirus B4- (CVB4-) induced diabetes was employed, showing that NK antiviral defence, raised by beta cells in response to IFNs, resulted in a reduced permissiveness to infection and subsequent natural killer (NK) cell-dependent death [59]. Another in vivo study (Figure 3(b)), using a T cell receptor transgenic model where T1D was induced via anti-CTLA-4 mAb treatment, revealed that higher frequency of NK cells exited in aggressive insulitis, resulting in b-islet cell destruction [60].

Conversely, there are several reports supporting a protective role exerted by NK cells, in NOD mice undergoing complete Freund's adjuvant (CFA). In this work, NOD/SCID mice immunized with CFA recover its protective effects when CD3^−^DX5^+^ NKs were adoptively transferred into animal recipients, by downregulating autoreactive T cell response [61] (Figure 3(c)).

Whether the murine model employed is relevant for the NK cell behaviour detected in the context of T1D is still debated. For example, NOD mice are characterized by an unusual genetic composition in the genomic regions that influence NK cell activity.

The NKG2D activatory receptor has been demonstrated to be overexpressed in NOD NKs due to the overexpression of its Rae-1 ligand. Further, diverse NK cell inhibitory receptors have been found to be differentially expressed in NOD mice as compared to C57BL/6 control animals [62]. Altogether, these genetic peculiarities may explain the low NKs activity detected in NOD mice [62, 63].

Moving to humans (Figure 3(d)), contrasting results have been reported as well (Figure 2). Most of them documented low number of circulating NK cells in T1D patients [64–66] or a functional altered state [67, 68]. The major concern regarding these studies is that, even if NK cells were directly isolated from T1D patients, the detection of functional alteration was performed by assessing NK cell cytolysis on K562 tumour cells.

Rodacki et al. investigated the frequency and activatory state of peripheral blood NK cells in individuals with T1D at different stages (recent versus long-standing onset) [69, 70]. No significant difference between the activatory state, as detected by IFN-*γ* and perforin release, was observed between NK cells derived from either recent or long-standing T1D patients. In contrast, lower expression of the NCR NKp30 and NKp40 was detected in NK cell isolated from long-standing type 1 as compared with control subjects. Further, gene expression analysis revealed that type 1 diabetic patients display an increased frequency of KIR gene haplotypes, including the activating KIR2DS3 gene, with a genetic interaction between the KIR and HLA complexes [69, 70].

## 4. Natural Killer Cells in Periodontitis

Periodontitis, defined as the inflammation of the periodontium involving the supporting tissues of the teeth, affects as much as 80% of the middle-aged population; by comparison, the prevalence of aggressive periodontitis reaches up to 1–1.5% [71].

The role of NK cells in periodontitis has been poorly investigated; however decreased Th cells and upregulation of NK cells during CP have been documented [72].

The role of NKs in periodontitis represents a still debated issue. Indeed, contrasting results have been reported by using human specimens. While some of them showed a relationship between NK number, phenotype, and periodontal state [73–76], others reported no significant correlation [73, 77].

Relevant increase of CD57 NK cells has been observed by Fujita et al. and related to periodontal diseases progression as a consequence of an unresolved immune response within periodontal tissues [73–76].

Contrast studies conducted by Fujita et al. and Cobb et al. revealed relatively low numbers of natural killer cells in chronic gingivitis and periodontitis samples, as compared to healthy subjects' correlation [73, 77].

**Figure 4:**
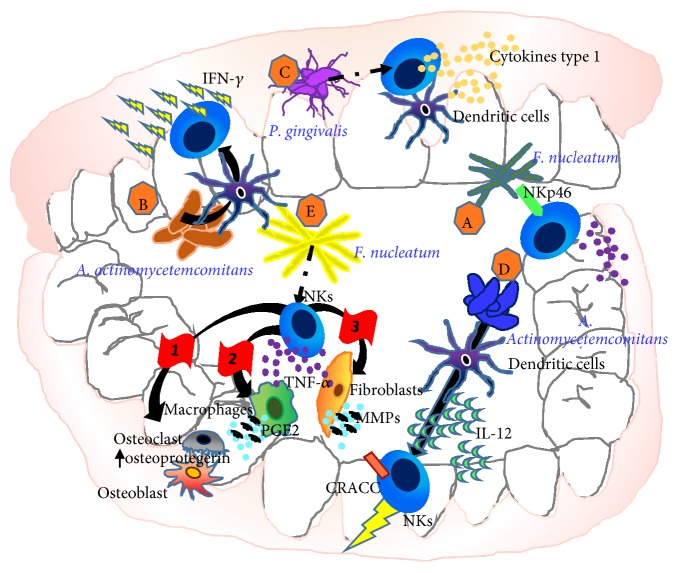
Interaction between NK cells and the main bacterial species involved in the pathogenesis of periodontitis. Direct recognition of* Fusobacterium nucleatum* through NKp46 worsts periodontal disease (A).* Actinobacillus actinomycetemcomitans* stimulate dendritic cells that in turn elicit rapid gamma interferon responses by natural killer cells (B).* P. gingivalis *induces a rapidly increase of type 1 cytokine production by both dendritic cells and NK cells (C).* Aggregatibacter actinomycetemcomitans i*nduces CD2-like receptor activating cytotoxic cells (CRACC) on NK cells, via activation of dendritic cells and subsequent interleukin 12 (IL-12) signalling that results in an aggressive (D). NK cell following* F. nucleatum recognition* can release TNF-*α* that on one hand leads to tissue damage by stimulating prostaglandin E2 release from monocytes and fibroblasts and secretion of metalloproteinases that degrade extracellular matrix (ECM) proteins and on the other hand induces osteoclast differentiation and activation by increasing RANKL expression and the suppression of osteoprotegerin, a cytokine receptor that belongs to tumour necrosis factor (TNF) receptor superfamily expression in osteoblasts, resulting in alveolar bone resorption (E).

Conversely, in vitro studies have focused on the interaction between NK cells and the main bacterial species involved in the pathogenesis of periodontitis (Figure 4), like* A. actinomycetemcomitans*,* P. gingivalis*, and* F. nucleatum* [78].

Direct recognition of* Fusobacterium nucleatum*, a gram-negative anaerobe microorganism ubiquitous to the oral cavity [79], by the NK cell natural cytotoxicity receptor NKp46, has been reported to aggravate periodontal disease [78] (Figure 4(A)).

*Actinobacillus actinomycetemcomitans*, a gram-negative bacterium which has been associated with severe oral infections [80], has been shown to elicit rapid gamma interferon responses by natural killer cells, via dendritic cell stimulation [81] (Figure 4(B)). Increased type 1 cytokine production by both dendritic cells and NK cells, following exposition to* P. gingivalis,* has been described, resulting in increased* P. gingivalis*-specific IgG2 [81] (Figure 4(C)).* Aggregatibacter actinomycetemcomitans, *a gram-negative anaerobic bacterium strongly associated with localized aggressive periodontitis [82], has been reported to indirectly induces CD2-like receptor activating cytotoxic cells (CRACC) on NK cells, via activation of dendritic cells and subsequent IL-12 signalling [83]. CRACC induction was reported to be more significantly pronounced in aggressive than chronic periodontitis and positively correlated with periodontal disease severity, subgingival levels of specific periodontal pathogens, and NK cell activation in vivo [83] (Figure 4(D)).

Other relevant mechanisms driving NK cell contribution to periodontitis involve ncr1 receptor recognition of still unknown ligands on* F. nucleatum* surface. This interaction resulted in TNF-*α* secretion that on one hand leads to tissue damage by stimulating prostaglandin E2 release from monocytes and fibroblasts, secretion of metalloproteinases that degrade extracellular matrix (ECM) proteins, and on the other hand induces osteoclast differentiation and activation by increasing RANKL expression and the suppression of osteoprotegerin, a cytokine receptor that belongs to tumour necrosis factor (TNF) receptor superfamily expression in osteoblasts, resulting in alveolar bone resorption [78] (Figure 4(E)).

Several conditions associated with periodontitis have been related to alteration in NK cells cytotoxicity. A part from those related to bacterial infections, T2DM [67], smoking habits [84] have been included as related conditions.

Fanconi anemia (FA), an autosomal recessive disorder characterized by progressive pancytopenia and congenital malformation of the skeleton [85], periodontitis, and gingivitis, represents common inflammatory states in patients with FA [85]. Natural killer (NK) cell numbers and function have been reported to be decreased in some FA patients and this was associated with impairment in the differentiation process of the NK cells subsets [85, 86]. Myers et al. showed perforin and granzyme reduced content in NK cells from children with FA as compared to controls [87].

Zeidel et al. reported that smokers without chronic obstructive pulmonary disease (COPD) showed impaired NK cytotoxic activity in peripheral blood and alteration in systemic production of pro- and anti-inflammatory cytokines [84].

A study performed on Papillon-Lefèvre syndrome (PLS), an autosomal recessive disorder that exited in aggressive periodontitis [88], revealed NK cell anergy, as compared to healthy subjects with impairment of NK cell cytotoxic function [89].

## 5. Natural Killer Cells in Atherosclerosis

Atherosclerosis is a chronic inflammatory disease affecting elastic and large muscular arteries that are characterized by lesions containing cholesterol, immune cells, smooth muscle cells, and necrotic cores. Macrophages, dendritic cells, and T cells represent the major immune cells populations within developing lesions, even if other immune cell components are involved, including NK cells [90]. Indeed, NKs have been observed within atherosclerotic plaques in humans as far as in mice [91, 92] and it has been demonstrated that in advanced atherosclerotic lesions they mostly localized in the necrotic core adjacent tissues, deep within plaques, and in shoulder regions [90].

**Figure 5:**
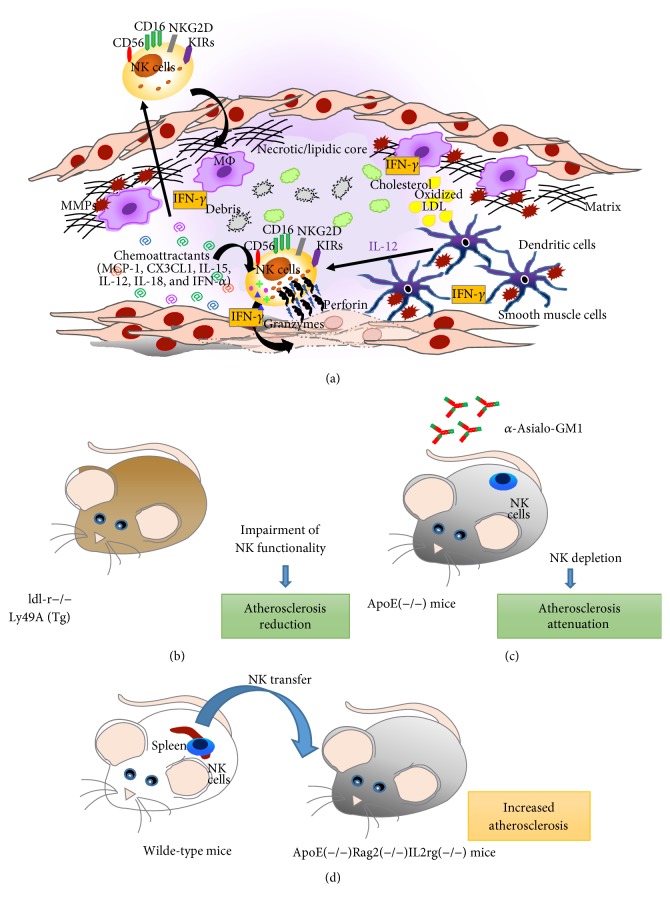
Proatherosclerosis role of natural killer cells. NK ability to induce atherosclerosis has been reported in several murine models and in humans. Several cytokines and chemokines within atherosclerotic lesions are supposed to promote NK recruitment towards atherosclerotic plaque, including monocyte chemoattractant protein-1 (MCP-1), fractalkine (CX3CL1), IL-15, IL-12, IL-18, and IFN-*α* that on one hand enhance NK cell migration and on the other hand induce NK activation resulting in an increased IFN-*γ* release. Moreover, these cytokines as far as oxidized LDL also promote the NK crosstalk with other immune cells, that is, dendritic cells and macrophages. DCs activated NKs by releasing IL-12 that in turn induce the production of IFN-*γ* by NKs that are able to lyse smooth muscle cells. In addition, IFN-*γ* released NK cells that in turn promote metalloproteinases (MMPs) secretion from cDCs and MΦ (a). In a chimeric atherosclerosis-susceptible low-density lipoprotein (LDL) receptor null (ldl-r−/−) mouse model, characterized by the impairment of NK cell functionality through the expression of a transgene encoding for Ly49A, it has been demonstrated that even if no difference in either serum total cholesterol concentrations or lipoprotein cholesterol distribution was observed between the two groups of mice, in Ly49A transgenic group, the deficiency of functional NK cells significantly reduced the size of atherosclerosis by 70% in cross-sectional analysis of the aortic root and by 38% in the intimal surface of the aortic arch (b). The administration of anti-Asialo-GM1 antibodies in ApoE(−/−) mice induces a NK cells depletion leading to an attenuation of atherosclerosis (c). The transfer of NK cells isolated from murine spleen into ApoE(−/−)Rag2(−/−)IL2rg(−/−) induces an enhancement of atherosclerosis (d).

It has been suggested that several cytokines and chemokines within lesions may be directly involved in NK cell recruitment towards atherosclerotic plaque. Among all, monocyte chemoattractant protein-1 (MCP-1) [93] as well as fractalkine (CX3CL1) has been shown as relevant cytokines able to enhance NK cell migration and activation resulting in an increased IFN-*γ* release [94]. In addition, IL-15, IL-12, IL-18, and IFN-*α*, which represent major NK cell chemoattractants, have been shown to promote atherogenic process, potentially activating NK cells, or promoting their crosstalk with other immune cells, including DC and monocytes/macrophages [95–97] (Figure 5(a)). NKs have been shown to participate in atherosclerosis via activatory receptors that recognize MHC-I molecules (MICA and MICB) [98] and by releasing IFN-*γ*, a proinflammatory cytokine [99]. In this context, it was demonstrated that oxidized low-density lipoprotein (LDL) can promote NK/dendritic cell crosstalk by activating the CD48-2B4 axis and inducing an increased production of IFN-*γ* by NKs [100] (Figure 5(a)).

To specifically confirm that NK cells infiltrate the vessel wall and contribute to atherosclerotic promotion and lesion development, several in vivo models have been employed. Whitman et al. create a chimeric atherosclerosis-susceptible LDL receptor null (ldl-r−/−) mouse model also characterized by the impairment of NK cell functionality through the expression of a transgene encoding for Ly49A. They demonstrated that, even if no difference in either serum total cholesterol concentrations or lipoprotein cholesterol distribution was observed between the two groups of mice, in Ly49A transgenic group the deficiency of functional NK cells significantly reduced the size of atherosclerosis by 70% in cross-sectional analysis of the aortic root and by 38% in the intimal surface of the aortic arch [92, 99] (Figure 5(b)).

Selathurai et al. demonstrated that treatments with anti-Asialo-GM1 in ApoE(−/−) mice resulted in NK cells depletion without affecting other lymphocytes ratios, associated with reduced atherosclerosis (Figure 5(c)). These effects have been shown to be independent from plasma lipids. Moreover NKs isolated from mouse spleens for adoptive transfer into lymphocyte-deficient ApoE(−/−)Rag2(−/−)IL2rg(−/−) mice confirmed the proatherogenic activity of NK cells. Further, the transfer of IFN-*γ*-deficient NK cells, but not granzyme B and perforin-deficient NK cells, resulted in an increased lesion size in the lymphocyte-deficient ApoE(−/−) mice as in wild-type NK cells. Necrotic core was increased by wild-type NK cells, whereas no changes were observed with perforin- and granzyme B-deficient NK cell transfer [98] (Figure 5(d)).

Cheng et al. showed that combined B, T, and NK cell deficiency accelerates atherosclerosis in BALB/c mice, demonstrating the impact of lymphocytes, including NKs, on lipoprotein metabolism along with the relevant contribution of lymphocyte subsets in plaque composition in atherosclerosis [101].

Recent studies have suggested that not only might the presence of NKs be considered in atherosclerosis progression, but also more importantly their ability to influence other immune cells should be evaluated. Several evidences demonstrated that NKs within atherosclerotic plaque are activated by dendritic cells. NK-released cytokines are able in turn to promote DC maturation, leading to an exacerbation inflammatory response. NK/DC crosstalk might be envisaged as a potential interaction occurring within atherosclerotic lesions, which might worsen disease progression [102] (Figure 5(a)). In fact, the crosstalk between activated dendritic cells/macrophages and NK cells induces IFN-*γ* release by NK cells that in turn promotes metalloproteinases (MMPs) secretion from cDCs and MΦ. Activated MΦ produce TNF-*α* increasing enhance endothelial cell adhesion molecules and MMPs can damage the extracellular matrix leading to atherosclerotic plaque destabilization [102] (Figure 5(a)).

Indirect evidence that NK cells might contribute to atherosclerotic disease is also provided by clinical observations in atherosclerotic patients. Recently, in a cohort of 124 patients it has been demonstrated that increased NK numbers were observed in the arm of the study including those patients with complications, suggesting NK cell direct contribution in atherosclerosis progression [103]. Similarly, in elderly atherosclerotic patients, an increased number of total circulating NKs characterized by an impaired cytotoxicity were shown [104]. According to these evidences, a significant reduction of CD56^dim^ NK cells and a concomitant loss of NK cell function in terms of cytolytic activity were also found in patients with unstable coronary artery disease (CAD) [105].

## 6. Conclusion

The contribution of natural killer cells to inflammatory disease insurgence and progression represent an intriguing topic for both basic scientists and clinicians. It is now clear that immune cell plasticity within the pathological microenvironment acts not only at local tissue but also at systemic levels. Several studies performed in different and distant pathologies like cancer, diabetes, and dental disorders that share an inflammatory state as a crucial hallmark showed altered NK cell activity at both local and systemic levels. This will be crucial to propose NK cells as potential circulating biomarkers to early detect diverse syndrome and/or predict further outcome.

Some studies, here discussed, supported the evidence that altered NK cell activities (enhanced/uncontrolled cytolysis versus impaired cytolytic functions) are associated with protective or deleterious effects. This knowledge suggests that further studies, requiring proper animal models and translation into human, are necessary to clarify the contribution of NK cells to the progression of inflammatory-related pathologies, aiming at identifying potential modulators able to shape NK cells, according to the pathological context. Finally, considering their direct and indirect (crosstalk with other arms on innate and adaptive immunity) contribution to inflammatory conditions, NK cells can be placed as relevant orchestrators in chronic diseases.

## Abbreviations

ADCC: Antibody-dependent cellular cytotoxicity
A-MuLV: Abelson murine leukaemia virus
CD: Cluster of differentiation
CFA: Complete Freund's adjuvant
COPD: Chronic obstructive pulmonary disease
CP: Chronic periodontitis
CRACC: CD2-like receptor activating cytotoxic cells
CTLA: Cytotoxic T-lymphocyte antigen
CVB4: Coxsackievirus B4
CX3CL1: Fractalkine
DC: Dendritic cells
dNK: Decidual NK cell
ECM: Extracellular matrix
FA: Fanconi anemia
FACS: Fluorescence-activated cell sorting
HLA: Human leukocyte antigen
IFN: Interferon
IL: Interleukin
ITAM: Immunoreceptor tyrosine-based activation motifs
ITIM: Immune tyrosine-based inhibitory motifs
KIR: Killer cell immunoglobulin-like receptor
LDL: Low-density lipoprotein
Ly49A: Killer cell lectin-like receptor subfamily A
M: Macrophages
MΦ: Naïve macrophages
MCP-1: Monocyte chemoattractant protein-1
MDSC: Myeloid-derive-suppressor cells
MHC: Major histocompatibility complex
MICA: MHC class I polypeptide-related sequence A
MICB: MHC class I polypeptide-related sequence B
MMPs: Metalloproteinases
miRNA: Micro-RNA
mRNA: Messenger RNA
N: Neutrophils
NCR: Natural cytotoxicity receptors
NK: Natural killer
NKG: Natural killer group
NOD: Nonobese diabetic
NOD/SCID: Nonobese diabetic/severe combined immunodeficiency
NSCLC: Non-small-cell lung cancer
PKA: Protein kinase A
PlGF: Placental growth factor
PLS: Papillon-Lefèvre syndrome
Rae: Retinoic acid early inducible
RANKL: Receptor activator of nuclear factor-*κ*B ligand
RMA-S: Rejected an MHC class I-deficient tumour cell line
SMAD: Small mother against decapentaplegic
STAT: Signal transducers and activators of transcription
Th: T helper
TANKs: Tumour associated natural killer cells
TINKs: Tumour infiltrating natural killer cells
TGF: Transforming growth factor
TME: Tumour microenvironment
TNF: Tumour necrosis factor
Treg: T regulatory
T1D: Type 1 diabetes
T2DM: Type 2 diabetes mellitus
v-abl: Abelson murine leukaemia viral oncogene homolog 1
VEGF: Vascular endothelial growth factor.
